# *Artemisia argyi* allelopathy: a generalist compromises hormone balance, element absorption, and photosynthesis of receptor plants

**DOI:** 10.1186/s12870-022-03757-9

**Published:** 2022-07-26

**Authors:** Jinxin Li, Tingting Zhao, Le Chen, Hong Chen, Dandan Luo, Changjie Chen, Yuhuan Miao, Dahui Liu

**Affiliations:** Key Laboratory of Traditional Chinese Medicine Resources and Chemistry of Hubei Province, Hubei University of Chinese Medicine, Wuhan, 430065 China

**Keywords:** Allelopathy, *Artemisia argyi*, Botanical herbicide, Photosynthesis, Phytohormone, Submicroscopic structure

## Abstract

**Background:**

Allelopathy is expressed through the release of plant chemicals and is considered a natural alternative for sustainable weed management. *Artemisia argyi* (*A. argyi*) is widely distributed throughout Asia, and often dominates fields due to its strong allelopathy. However, the mechanism of *A. argyi* allelopathy is largely unknown and need to be elucidated at the physiological and molecular levels.

**Results:**

In this study, we used electron microscopy, ionomics analysis, phytohormone profiling, and transcriptome analysis to investigate the physiological and molecular mechanisms of *A. argyi* allelopathy using the model plant rice (*Oryza sativa*) as receptor plants. *A. argyi* water extract (AAWE)-treated rice plants grow poorly and display root morphological anomalies and leaf yellowing. We found that AAWE significantly inhibits rice growth by destroying the root and leaf system in multiple ways, including the integrity of ultrastructure, reactive oxygen species (ROS) homeostasis, and the accumulation of soluble sugar and chlorophyll synthesis. Further detection of the hormone contents suggests that AAWE leads to indole-3-acetic acid (IAA) accumulation in roots. Moreover, ionomics analysis shows that AAWE inhibits the absorption and transportation of photosynthesis-essential mineral elements, especially Mg, Fe, and Mn. In addition, the results of transcriptome analysis revealed that AAWE affects a series of crucial primary metabolic processes comprising photosynthesis in rice plants.

**Conclusions:**

This study indicates that *A. argyi* realizes its strongly allelopathy through comprehensive effects on recipient plants including large-scale IAA synthesis and accumulation, ROS explosion, damaging the membrane system and organelles, and obstructing ion absorption and transport, photosynthesis and other pivotal primary metabolic processes of plants. Therefore, AAWE could potentially be developed as an environmentally friendly botanical herbicide due to its strong allelopathic effects.

**Supplementary Information:**

The online version contains supplementary material available at 10.1186/s12870-022-03757-9.

## Background

Allelopathy refers to the chemical interactions between all organisms (including plants, algae, bacteria, and fungi) with their environment through the medium of chemical substances. To date, the most studied allelopathic effect is plant-plant allelopathy, which refers to the beneficial or harmful effects of plants on the growth and development of neighboring plants via the release of specific secondary metabolites into the environment [1]. The effects of plant-plant allelopathy are mainly reflected in niche competition for growth-limiting resources, including space, light, water, and nutrients. In this regard, the "Novel Weapons Hypothesis" [2] has been proposed to explain the dominant ecological niches of invasive plants. Alien plants synthesize allelochemicals and release them into the local community, which can inhibit the growth of native plants by promoting the expansion of invasive species and conferring upon them a dominant position during the competition for survival. In addition, some native plants with strong allelopathy can also become dominant communities in various ecological environments through competition. Based on allelopathic theory, allelopathy involves the synthesis of allelochemicals, which can be used as natural pesticides. Currently, many allelochemicals have been developed as herbicides [3], bacteriostasis [4], and algal inhibitory agents [5], representing new methods for biological controls in agricultural ecosystems and marine ecosystems. Jabran et al. [6] demonstrate that 34% of yield losses of major food crops worldwide are caused by weeds, which can significantly affect the growth and development of crops and are more harmful than diseases and insect pests. Compared to chemical herbicides, applying allelopathic practices during weeding can avoid environmental pollution and the genetic variation of weed resistance. Therefore, many attempts have been made to introduce allelopathy into weed management, including allelopathic straw mulching, stubble cultivation, and the development of plant-derived herbicides. Based on the advantages of biodegradation after entering the environment [7], allelochemical-derived plant herbicides have less pollution, protecting agricultural production [8], and alleviating social health problems. Many allelochemicals can be developed as highly effective plant-derived herbicides for weed control after preparing dosage forms or selecting the appropriate application timing. For example, Uddin et al. [9] identified a hydrophobic compound from sorghum as a wettable powder that can suppress weed growth. Cook [10] isolated strigolactone from cotton root secretions that effectively induced parasitic weeds such as *Striga asiatica* and *Orobanche coerulescens* to "suicide germination" in the absence of a host. Therefore, botanical herbicides can replace chemical herbicides in weed prevention.

Allelochemicals are the carriers of plant allelopathy and are produced by different plants. Allelochemicals play different roles, which leads to the complexity and diversity of plant allelopathy mechanisms. These mechanisms include blocking membrane permeability, water and nutrient uptake, respiration, photosynthesis, protein and nucleic acid synthesis, and growth regulation in tested plants [11]. For example, treating the root tuber extract of *Ipomoea batatas* significantly increases the malonaldehyde (MDA) content of *Alternanthera philoxeroides* new leaves and deepens damage to the cell membrane [12]. In addition, phthalic acid, dibutyl phthalate, and diphenylamine affect the absorption of N, P, and K by tomato seedling roots [13], and p-hydroxybenzoic acid can significantly inhibit the absorption of K^+^, NO_3_^−^ and H_2_PO_4_^−^ by cucumber roots [14]. Moreover, Ade et al. [15] reported that treating *Dysphania ambrosioides* volatile oil makes the chloroplasts of *Vicia faba* scatter from the cell wall and disperse in the center of the cell, resulting in the disappearance of starch grains and the hinders of photosynthesis. Li et al. [16] demonstrated that a high concentration of *Cerasus sachalinensis* root extract downgrades the respiration rate of seedling roots and reduces oxygen uptake of oxygen by the roots. Meanwhile, the extract also improves the activities of glucose-6-phosphate dehydrogenase (G-6-PDH) and 6-phosphogluconate dehydrogenase (6-G-PDH), regulates the large-scale operation of PPP, and blocks EMP-TCA, leading to seedling respiratory and metabolic disorders.

*Artemisia argyi* (*A. argyi*) is a famous medicinal plant primarily distributed in eastern Asia. It has dual functions as a medicine and food, and is widely used in medical treatments and dietotherapy. In addition, modern medical and pharmaceutical studies have shown that *A. argyi* contains volatile oils, flavonoids, terpenoids, phenylpropanoids, organic acids, and polysaccharides [17], which have bacteriostatic, nephrotoxic, antioxidative, anti-insect, and anticancer functions [18]. This study provides a broader direction and suggestions for the development and utilization of *A. argyi*. In our previous study, we found that *A. argyi* had a strong allelopathic inhibitory effect on various weeds, which was demonstrated in incubator environments, pot conditions, and field experiments [19]. We also found that the *A. argyi* water extract (AAWE) had stronger herbicidal activity than other extracts. AAWE is rich in phenolic acid components such as caffeic acid, cryptophyllic acid, neochlorogenic acid, isochlorogenic acid A, and chlorogenic acid [19], which is consistent with the phenomenon that phenolic acid components have stronger inhibitory effects on weeds [20]. However, the mechanism underlying AAWE allelopathy was unclear, so this research conducted a systematic study. After AAWE treatment, we assessed whether the cellular structure of recipient plants was disrupted, how the relevant physiological indicators were affected, and what changes occurred in the expression profile of the recipient plants. Therefore, it is necessary to find a suitable model plant for mechanistic studies at the physiological and molecular levels. As a recognized model plant, rice (*Oryza sativa*) has been widely used to analyze physiological mechanisms under a variety of stresses, including zinc deficiency [21], phosphorus deficiency [22], nitrogen deficiency [23], high temperature [24], low temperature [25], and drought and salt stress [26]. Therefore, it is suitable to select rice as the test plant to explore the allelopathic mechanism of *A. argyi*.

In this study, we demonstrated that the allelochemicals of *A. argyi* can lead to massive auxin synthesis and accumulation in rice. Moreover, this allelopathy caused the explosion of reactive oxygen species (ROS), the destruction of the membrane system, damage to organelles, and the further inhibition of photosynthesis, carbon assimilation, and starch formation in receptor plants. In addition, allelochemicals can destroy the root tip cells of rice, which leads to a failure in the absorption and transmission of plant nutrient elements. Ultimately, allelochemicals result in rice tissue decline, plant senescence, and death. Our results provide important insights into the regulatory mechanism of *A. argyi* allelopathy and the development of new plant-derived herbicides.

## Results

### Inhibition effects of *A. argyi* water extract (AAWE) on rice seedlings

**Fig. 1:**
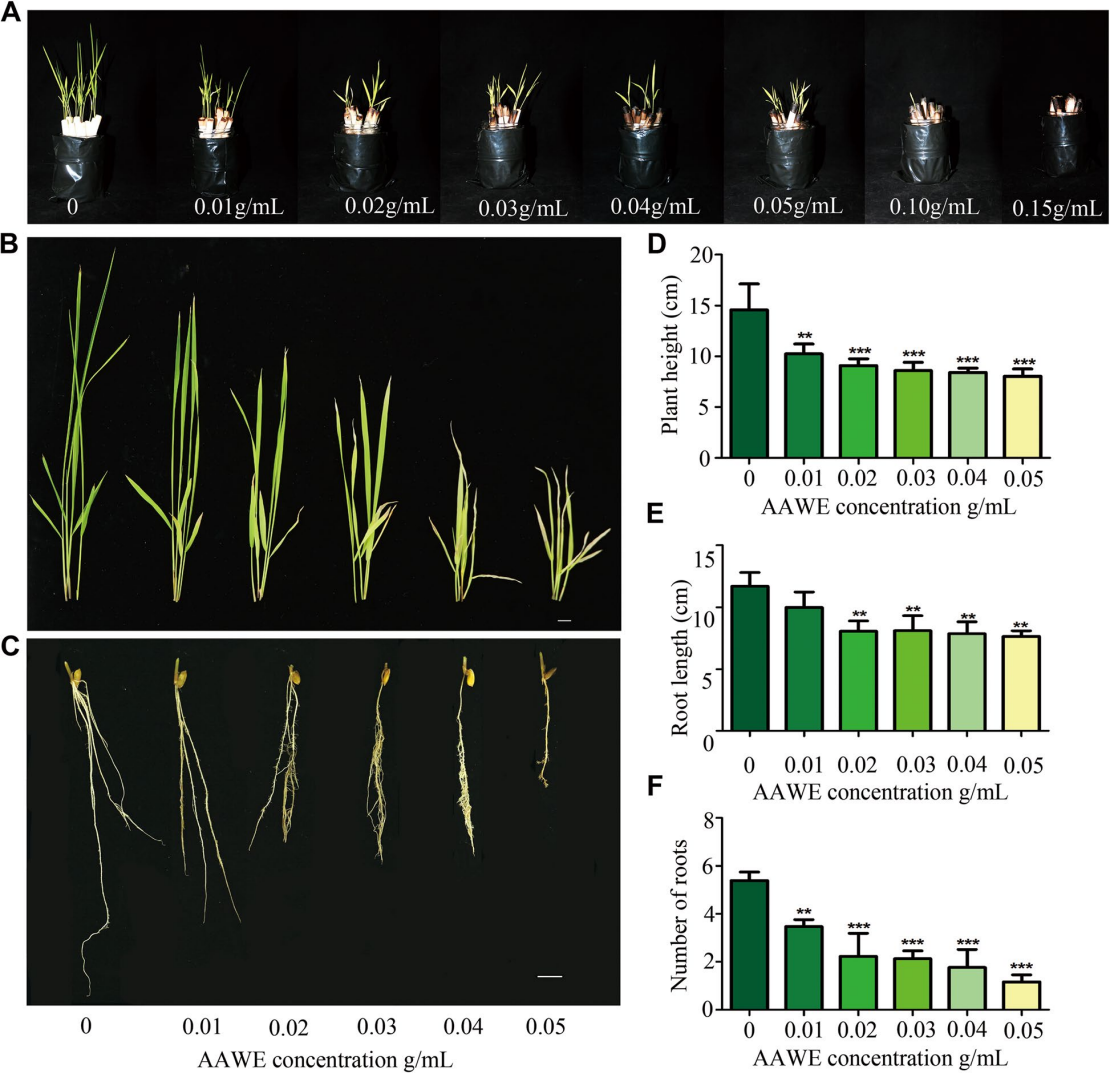
Germination situation and morphological features of rice treated with AAWE. **A** Germination and growth situation of rice in a culture flask. **B** Plants showing lesions on the aboveground part at lower concentrations. Scale bar = 1 cm. **C** The underground parts of plants showed inhibition and abnormal morphological structure at lower concentrations. Scale bar = 1 cm. (**D**), (**E**), and (**F**) represent the plant height, root length, and root number of different treatment groups, respectively. Values are the means ± SD for three biological replicates (**P < 0.01, ***P < 0.001)

In our previous study, we found that AAWE strongly inhibited the seed germination and growth of *Brassica pekinensis*, *Lactuca sativa*, *Oryza sativa*, *Portulaca oleracea*, *Oxalis corniculata,* and *Setaria viridis* [19]. To further explore the mechanisms underlying the allelopathic inhibition of AAWE, we first investigated the site of action using rice as a model plant. Our results showed that AAWE had a concentration-dependent inhibitory effect on rice (Fig. 1A). Plant growth could be significantly inhibited at a very low extract concentration (0.01 g/mL), while the rice seeds could not germinate when the AAWE treatment concentration was up to 0.15 mg/mL (Fig. 1A). Therefore, to investigate the influence of AAWE treatment on plants, we chose the lower treatment concentration range (0.01–0.05 mg/mL) at which rice could germinate and grow. As shown in Figs. 1B and 1C, rice growth was significantly (*P* < 0.01) inhibited under the 0.01 g/mL AAWE treatment, and the inhibitory effect was enhanced as the treatment concentration increased. In addition, the AAWE-treated rice leaves yellowed. Further statistical analysis showed that the plant height, root length, and number of roots under AAWE treatment significantly decreased compared to the control (Fig. 1D, E, F). These results indicate that AAWE dramatically inhibited the root and leaf system of rice. Therefore, we further investigated the mechanisms of AAWE inhibitory effects on the development of rice roots and leaves.

### AAWE destroyed the growth and development of the root system

**Fig. 2:**
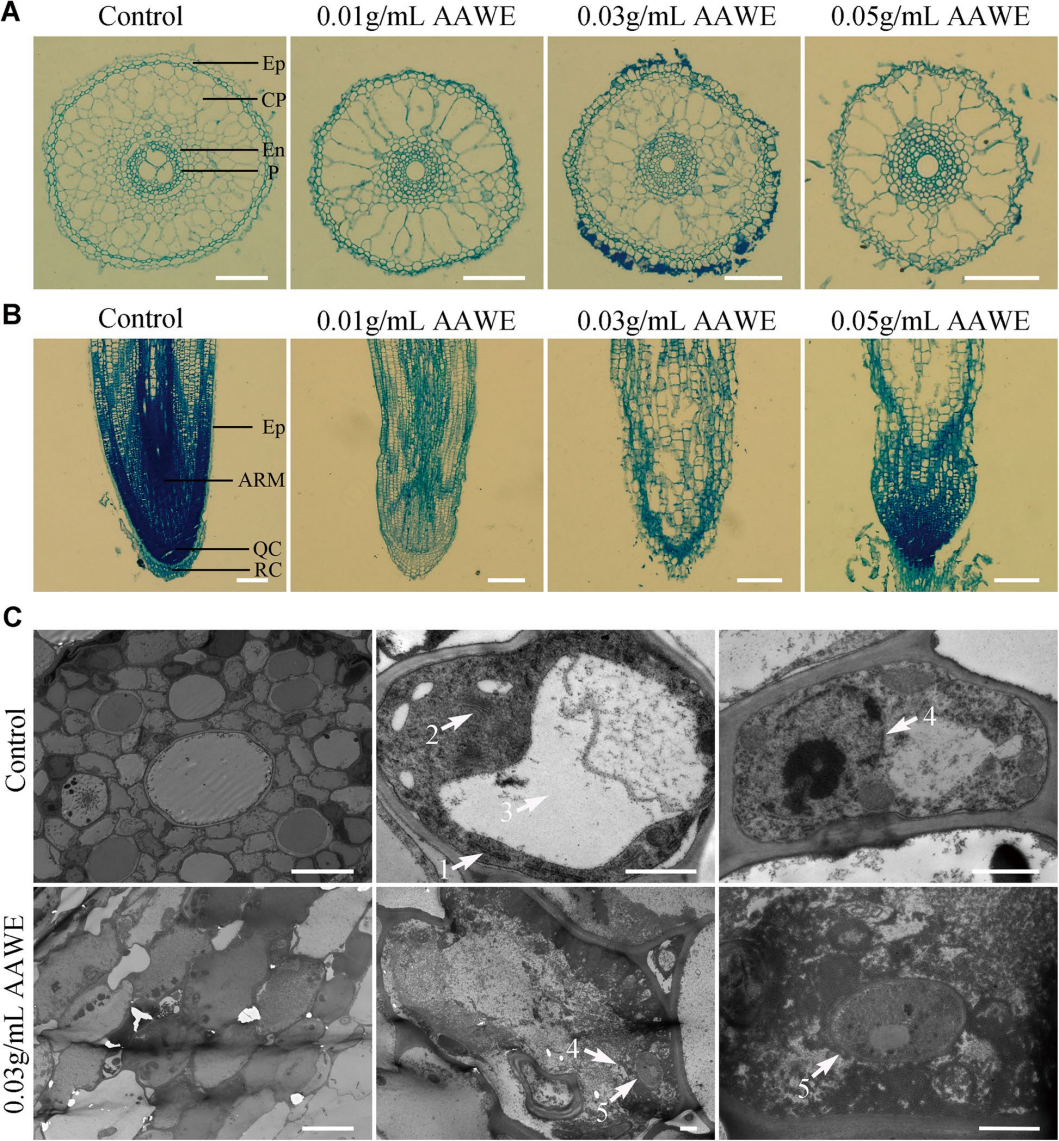
AAWE-treated rice plants have defects in the growth and development of root tip cells. **A** Microstructure of root tip cells by observing transection. Ep, epidermal layer; CP, cortical parenchyma; En, endothecium; P, pericycle. Scale bars = 100 μm. **B** Microstructure of root tip cells by observing longitudinal sections. Ep, epidermal layer; AM, apical meristem; QC, quiescent centre; RC, root cap. Scale bars = 200 μm. **C** Transmission electron microscopy of control and AAWE-treated root tip cells in rice. Two left panels, middle panels, and right panels indicate apical sections, single cells, and nuclei, respectively. 1, endoplasmic reticulum; 2, Golgi body; 3, vacuole; 4, nucleus; 5, fungi. Scale bars: 10 μm, 10 μm, and 1 μm (the first row runs from left to right); 10 μm, 1 μm, and 1 μm (the second row runs from left to right)

As shown in Supplemental Fig. 1A, the rice root number decreased, and the structure significantly changed after AAWE treatment compared to the control. We performed microscopic observation of transverse sections of the control and AAWE-treated rice root tip cells to investigate the influence of AAWE. In the control, the cells in the transected surface of the root tip of rice seedlings were regularly and uniformly arranged and were composed of clearly distinguishable cell layers. In order from the outside to the inside, these were the epidermal layer, cortical parenchyma, endothecium, and pericycle. However, after AAWE treatment, the number of cell layers in the rice root tips decreased, and the arrangement was disordered. Moreover, most of the cortical parenchyma cells expanded more than twice, indicating that the root tip cells probably lost their ability to divide (Fig. 2A). This effect was enhanced as AAWE treatment concentrations increased, and epidermal cells began to shed at 0.03 g/mL (Fig. 2A). The results of longitudinal section observation were consistent with transverse section observations, the root tip cells of the control seedlings were arranged regularly and clearly, and the cell classification in each functional area was obvious. Nevertheless, epidermal cells in the rice root tip became wrinkled, shrunken, and even ruptured when exposed to 0.01 g/mL AAWE. The cells in the shoot apical meristem, quiescent center, and root cap were also disordered (Fig. 2B). In addition, scanning electron microscope (SEM) analysis demonstrated that aberrant alterations occurred along the surface of the roots upon introduction to AAWE compared to the control (Supplemental Fig. 1B).

**Fig. 3:**
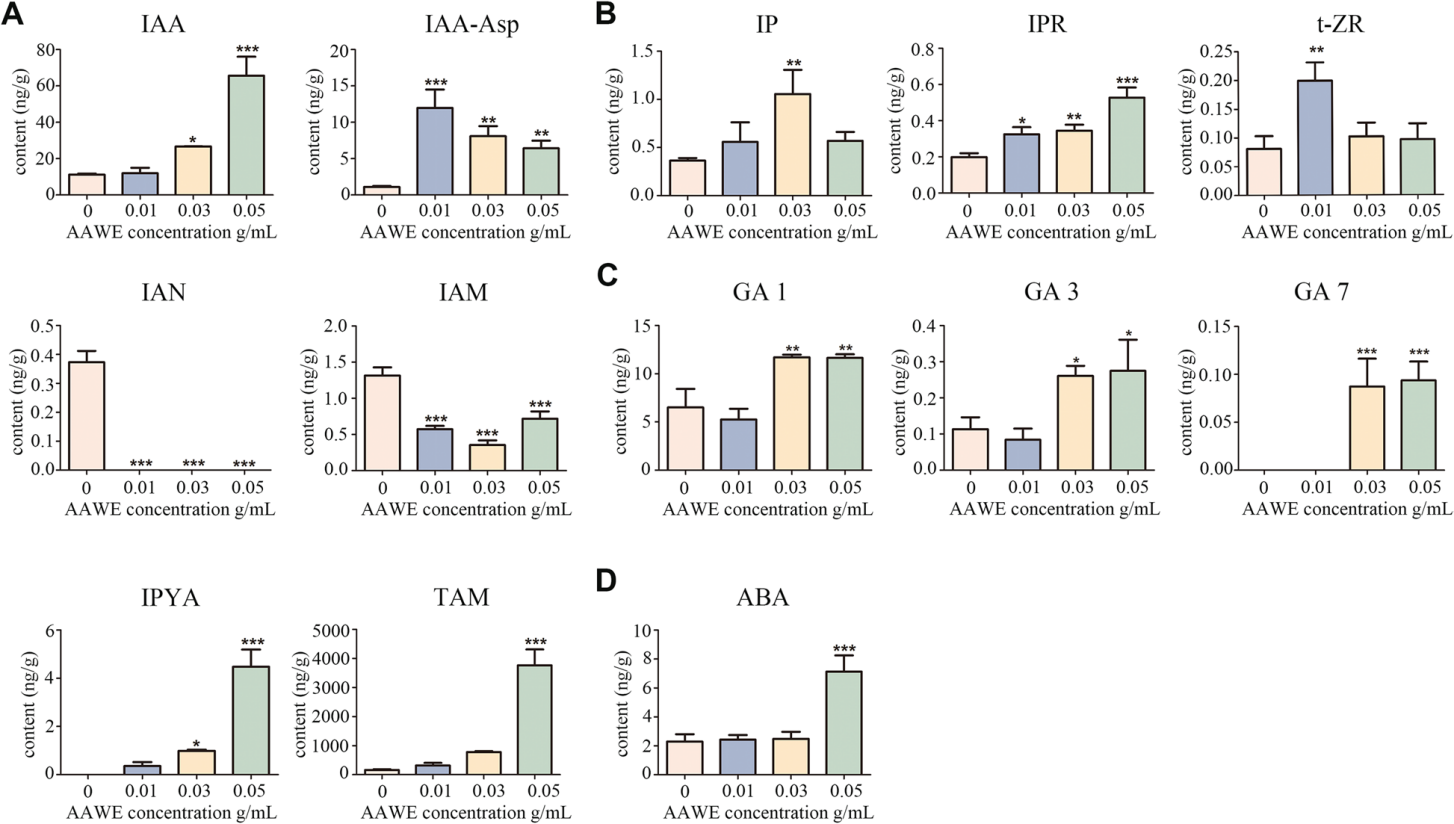
Phytohormone contents in the rice root system significantly changed under AAWE treatment. **A** The contents of auxins and intermediates in different IAA synthesis pathways. **B** The contents of cytokinins. **C** The contents of gibberellin. **D** The contents of abscisic acid. Values are the means ± SD of three biological replicates (*P < 0.05, **P < 0.01, ***P < 0.001)

**Fig. 4:**
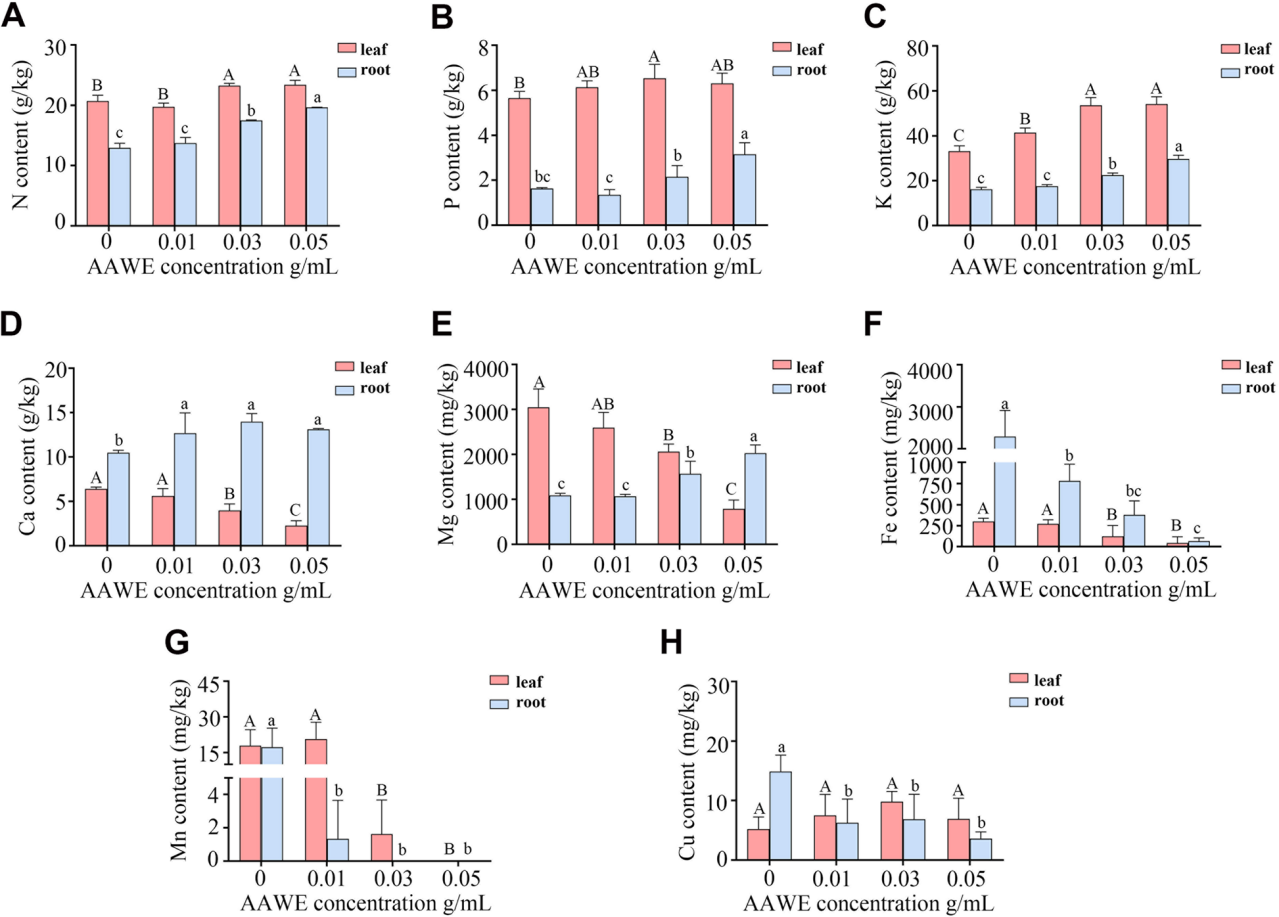
AAWE inhibited the absorption and transportation of photosynthesis-essential mineral elements in rice. **A-H** The contents of N (**A**), P (**B**), K (**C**), Ca (**D**), Mg (**E**), Fe (**F**), Mn (**G**), and Cu (**H**), respectively. The red columns represent the contents of elements in the leaves, and the blue columns represent the content of elements in the roots. Values are means ± SD of three biological replicates. Different letters in the same tissue indicate significant differences (*P* < 0.05)

**Fig. 5:**
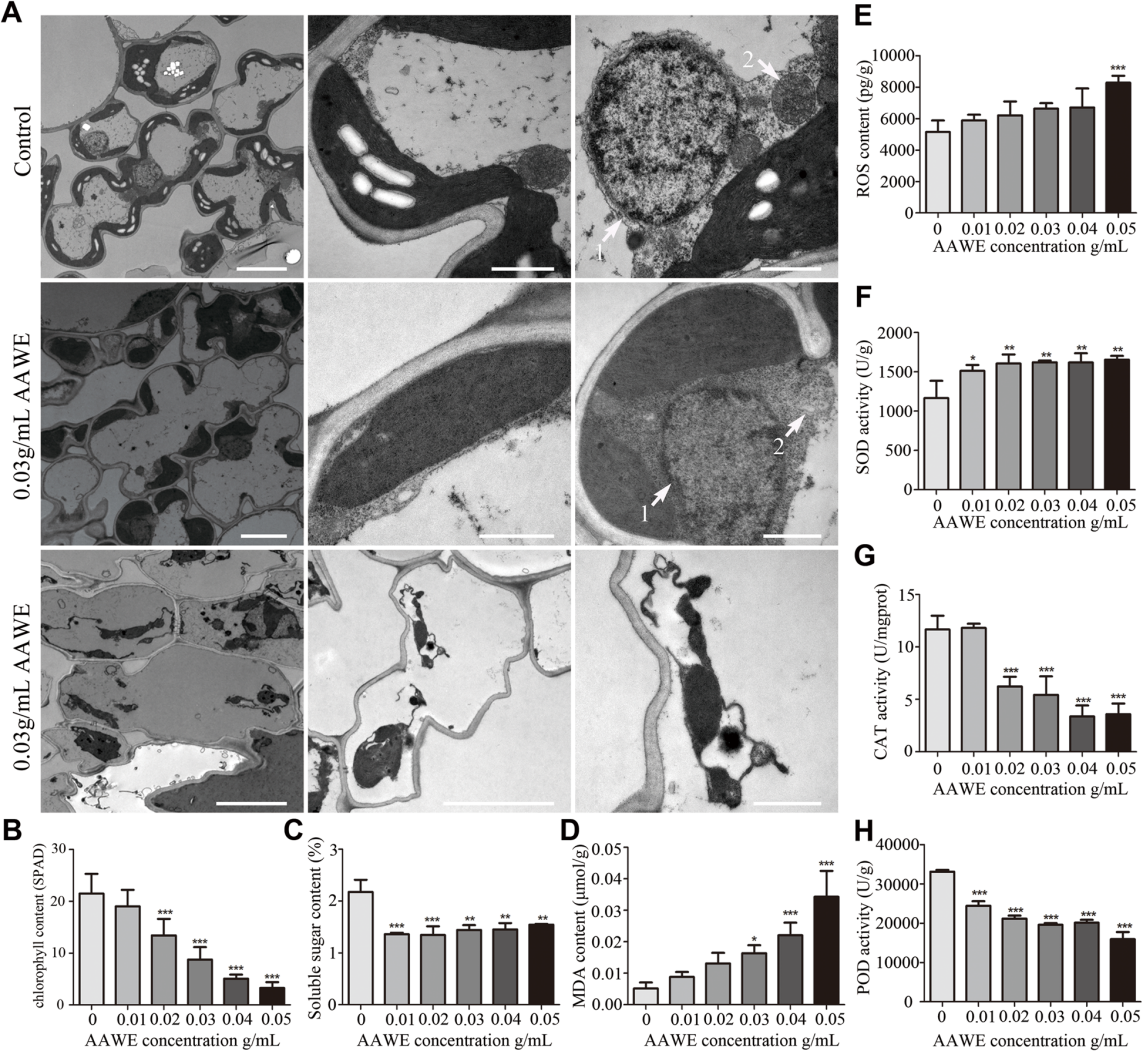
AAWE destroyed the growth and development of rice leaves. **A** Transmission electron microscopy of control and AAWE-treated mesophyll cells. The first row (from left to right): single mesophyll cell, chloroplast structure, nucleus, and mitochondrial structure of the control, respectively. Scale bars: 5 μm, 1 μm, 1 μm (from left to right). The second row (from left to right): AAWE-treated single mesophyll cell, chloroplast structure, nucleus, and mitochondrial structure, respectively. Scale bars: 5 μm, 1 μm, 1 μm (from left to right). The third row: cell membrane of AAWE-treated mesophyll cell. The left, middle, and right panels represent different magnifications. Cell membrane was damaged in the AAWE-treated mesophyll cell. Scale bars: 2 μm, 5 μm, 1 μm (from left to right). **B** Detection of chlorophyll content in the control and AAWE-treated rice leaves. The chlorophyll content in the middle part of the outermost leaves was directly measured with a chlorophyll meter, and the measured results were indicated by the SPAD value of the instrument reading. The higher the SPAD value indicates, the higher the chlorophyll content (***P < 0.001). **C** Soluble sugar content in the control and AAWE-treated rice leaves (**P < 0.01, ***P < 0.001). **D-E** AAWE caused oxidative damage in rice leaves. D, MDA content; E, ROS content (*P < 0.05, **P < 0.01,***P < 0.001). **F–H** Antioxidant enzyme activity in the control and AAWE-treated rice leaves, including SOD (**F**), CAT (**G**), and POD (**H**) (*P < 0.05,**P < 0.01,***P < 0.001)

We further performed transmission electron microscopy (TEM) observations to compare the subcellular structure of control and AAWE-treated rice plants. The results showed that cells in the blank group were regularly and closely arranged, while cells in the treatment group (0.03 g/mL) were enlarged and disordered (Fig. 2C). Magnified observation of a single cell revealed that the membrane system of the blank group was complete, with clearly visible endoplasmic reticulum (Fig. 2C-1), Golgi body (Fig. 2C-2), vacuoles (Fig. 2C-3), and other organelles. However, organelles could hardly be found, and many black deposits appeared inside the cell under AAWE treatment, indicating that the organelles probably degraded. We further compared the nuclear structure in the blank and AAWE treatment groups to detect whether the control center of the whole cell was affected. The results showed that the nucleus in the blank group was intact, but the nuclear membrane of the treated group was degraded, and the nucleoli diffused (Fig. 2C-4). Fungi were also observed in the AAWE treatment group, including the nucleus (Fig. 2C-5), which could be due to very weak antifungal activity after AAWE treatment. These results demonstrated that AAWE had a strong destructive influence on the integrity of organelles and nuclei in rice cells.

### AAWE altered phytohormone homeostasis in rice roots

Phytohormones, including auxin, cytokinin, gibberellins, and abscisic acid, play vital roles in plant root development [27]. As rice roots were destroyed after AAWE treatment, we explored whether the regulation of endogenous phytohormones was out of balance. Therefore, we detected the content of endogenous phytohormones in the control and AAWE-treated roots. For auxins, 0.05 g/mL AAWE treatment promoted the production of the main auxins (IAA), and the IAA content in rice roots reached 5.83-fold that of the control. Meanwhile, as a low molecular weight binding state of IAA, IAA-ASP content increased remarkably under low AAWE concentration treatment. Although the IAA-ASP content showed a decreasing trend as the AAWE treatment concentration increased, it still presented a significant enhancement compared to the blank control group. These results suggest that AAWE promotes auxin production and accumulation of recipient plants (Fig. 3A).

For cytokinins, one free cytokinin (isopentenyladenosine, IP) and two tRNA cytokinins (iP Riboside, IPR and trans-zeatin riboside, t-ZR) were determined. The results showed that AAWE had different effects on different cytokinins (Fig. 3B). The IP and t-ZR contents first increased and then decreased as the AAWE treatment concentration increased. The IP content was highest at 0.03 g/mL, while that of t-ZR was at 0.01 g/mL, which were 2.90 and 2.47 times those of the control, respectively. In addition, the IPR content in rice roots was significantly greater than that in the blank control, while the IPR content increased in an AAWE dose-dependent manner. At the maximum concentration of AAWE (0.05 g/mL), IPR accumulation reached 0.53 ng/g, which was approximately 2.66 times greater than the level of IPR in the blank control.

We further measured three physiologically active gibberellins to evaluate the effect of AAWE on gibberellin content. The results revealed that gibberellin showed an increasing trend under AAWE stress (Fig. 3C)*.* GA_1_ and GA_3_ significantly increased when the treated AAWE concentration reached 0.03 g/mL. GA_7_ began to be synthesized and detected under the 0.03 g/mL AAWE treatment, although it was barely synthesized by plants under natural conditions and low AAWE treatment concentrations. In addition, the level of ABA in 0.05 g/mL AAWE-treated rice roots was 3.10 times higher than that in the control, but low-concentration AAWE treatment did not significantly impact ABA content (Fig. 3D). This suggests that the ABA pathway was not particularly sensitive to AAWE. In summary, rice hormones in roots showed an upward trend after AAWE treatment, and the contents of some hormones reached several times of those in the blank group, leading to negative effects on normal plant growth.

### AAWE inhibited the absorption and transportation of photosynthesis-essential mineral elements

The root is the main organ of plants that absorbs mineral nutrients, which are transported to the aboveground parts, especially the leaves. The uptake and transportation of mineral nutrients are essential for plant life activities. We next measured essential elements in rice roots to investigate the impact of AAWE on mineral nutrient uptake and transportation. The N, P, and K contents in both leaves and roots were up-regulated slightly under AAWE treatment (Fig. 4A, B, C). These results indicated that *A. argyi* allelopathy did not affect the absorption of macroelements and likely exhibited a promotion effect. Ca and Mg play significant roles in maintaining cell integrity [28]. Mg participates in the synthesis of chlorophyll and maintains the stability of the chloroplast structure [29]. Our results showed that the contents of Ca and Mg in AAWE-treated rice plants were significantly enriched in roots but decreased in leaves (Fig. 4D, E), indicating that the transport of these two nutrient elements from roots to leaves was mostly blocked. More importantly, the absorption of Fe and Mn was significantly inhibited in roots and leaves, and Mn was barely absorbed by the plants under high concentrations of AAWE treatment (Fig. 4F, G). Due to the crucial roles of Fe and Mn in photosynthesis [30, 31], the sharp decrease of these two elements in leaves and roots indicated that the photosynthetic efficiency of rice treated with AAWE likely decreased. Finally, Cu, which promotes photosynthesis and protein accumulation in plants [32], had no significant difference between control and AAWE-treated rice plants in leaves but significantly decreased in AAWE-treated roots compared to the control (Fig. 4H). Altogether, AAWE treatment mainly affected the absorption and transport of trace elements, which likely led to defects in photosynthesis and the primary metabolism of rice.

### AAWE destroyed the growth and development of leaves

In addition to abnormalities in roots, we found that AAWE treatment also seriously inhibited the growth of rice leaves. The AAWE-treated rice leaves exhibited abnormal symptoms, such as leaf rolling, yellowing, and withering (Supplemental Fig. 2). We used TEM to observe ultrastructural changes in AAWE-treated rice leaf cells. In the control group, mesophyll cells had an obviously intact structure and chloroplasts with abundant starch granules attached to the cell wall (Fig. 5A). However, in the AAWE-treated group, anomalous black sediment was observed in the cells, the number of chloroplasts was significantly reduced, and no starch grains were observed (Fig. 5A). Moreover, chloroplasts in AAWE-treated rice were significantly different from those in the control. In the control, chloroplasts presented a long fusiform structure with a clear and complete membrane structure. The matrix in the chloroplasts was uniform and dense, and grana thylakoids in the control were orderly stacked. Compared to the control, the membrane structure of AAWE-treated chloroplasts was obviously damaged, the arrangement of the internal grana and thylakoid was disordered or even disappeared, and the lamellae were blurred and loosely arranged, which probably led to the absence of starch grains (Fig. 5A). Further analysis showed that the chlorophyll content in 0.02 g/mL, 0.03 g/mL, 0.04 g/mL, and 0.05 g/mL AAWE-treated rice leaves significantly decreased compared to the control. And the inhibitory effect of AAWE on chlorophyll content showed a dose-dependent relationship with AAWE concentration. The chlorophyll content in 0.05 g/mL AAWE-treated rice leaves only accounted for 15.34% of that in the blank control, indicating that photosynthesis was almost completely lost (Fig. 5B). Moreover, the soluble sugar content decreased under *A. argyi* allelopathy (Fig. 5C). These results suggest that AAWE treatment affects chlorophyll content and photosynthesis, leading to a decrease in photosynthetic products.

Another interesting phenomenon is that almost the entire membrane structure was significantly damaged in AAWE-treated mesophyll cells. The nuclear membrane of the blank group was clear, and the chromatin was evenly distributed in the nucleus, while the membrane structure of the AAWE-treated group was dissolved (Fig. 5A-1). Mitochondria in the blank group had obvious membrane boundaries, while the boundary between mitochondria and other organelles was blurred in the AAWE-treated group (Fig. 5A-2). In addition, the cell membrane was damaged, scattered in the center of the cell, and no longer close to the cell wall (Fig. 5A). These results demonstrate that AAWE treatment seriously destroys the membrane structure and the integrity of organelles in rice mesophyll cells.

### AAWE caused oxidative damage in rice leaves

Previous studies have shown that the intracellular level of reactive oxygen species (ROS) dramatically increases in harsh environments, which seriously damages the cell structure. Considering the severely damaged cell structure, we speculated that an ROS burst likely occurred in AAWE-treated rice mesophyll cells. Therefore, we detected ROS levels in both the control and AAWE-treated groups. Consistent with our expectations, AAWE treatment induced an ROS burst in rice. ROS levels increased as the AAWE concentration raised. The ROS content reached 8280 pg/g in 0.05 g/mL AAWE-treated rice, which was much higher than the 5145 pg/g in the control (Fig. 5E). The MDA content is usually used as an indicator to assess the extent of lipid peroxidation and membrane system damage. Therefore, we further measured the MDA content in the control and AAWE-treated groups. The results showed that the MDA content in rice seedlings also continuously increased as the AAWE concentration increased (Fig. 5D). The MDA content in rice under the 0.05 g/mL AAWE treatment was 5.68 times that of the control. These results suggest that AAWE greatly induced ROS bursts and MDA accumulation in rice.

Plants have evolved many antioxidative enzymes to eliminate ROS, including superoxide dismutase (SOD), peroxidase (POD), and catalase (CAT), which can protect cells from damage by scavenging or weakening the toxic effects of free radicals [33]. We found that the activities of these three enzymes were greatly affected by AAWE treatment. All AAWE-treated samples displayed higher SOD activities than the control samples, which were approximately 37.82% ~ 41.94% (*P* < 0 0.01) higher than those of the corresponding controls (Fig. 5F). In contrast, POD activity showed a concentration-dependent decrease from 0.01 to 0.05 g/mL (*P* < 0.001). POD activity in 0.05 g/mL AAWE-treated samples was only 48.09% that of the control group (Fig. 5G). Similarly, CAT activity also decreased after AAWE treatment (Fig. 5H). This suggests that under low-stress conditions, cells can activate their own antioxidant defense system and quickly clear their own ROS. However, when the stress concentration exceeds its own tolerance, oxidative damage will be very serious.

### AAWE strongly influenced the transcription of key driver genes related to primary metabolism

**Fig. 6:**
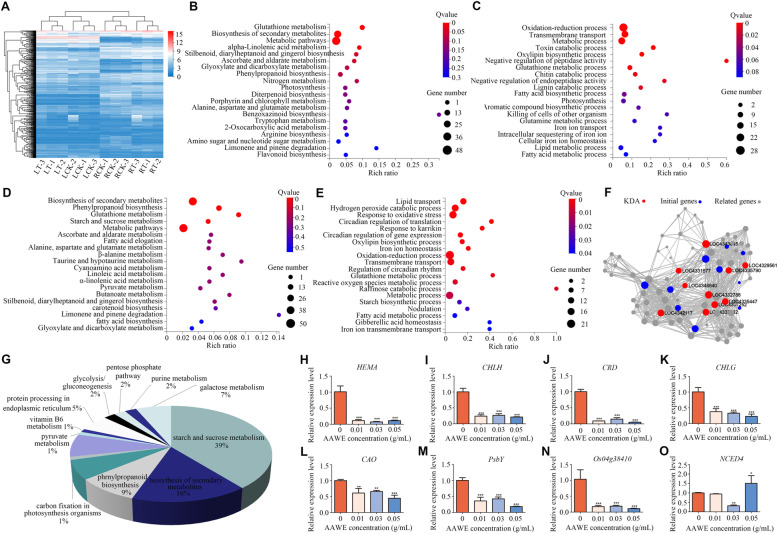
Differentially expressed genes between the control and AAWE-treated rice plants. **A** Hierarchical cluster analysis of the significantly changed DEGs (|Log2FC|≥ 1). The color key represents FPKM-normalized log2-transformed counts. **B** Top 20 KEGG pathway enrichment analysis of DEGs in leaves. The colors are shaded according to the Q-value level, as shown in the color bars gradually from low (red) to high (blue); the size of the circle indicates the number of DEGs from small (less) to large (more). **C** GO enrichment analysis of leaf DEGs involved in molecular processes. **D** Top 20 KEGG pathway enrichment analyses of DEGs in roots. **E** GO enrichment analysis of root DEGs involved in molecular processes. **F** Key driving gene analysis of DEGs in the whole rice plant after AAWE treatment. The genes labeled red are the key driver genes. **G** Functional annotation analysis of related genes connected with key driver genes. **H-L** Relative expression levels of chlorophyll biosynthesis genes involved in the porphyrin and chlorophyll metabolism pathways. **M-N** Relative expression levels of genes involved in the photosynthesis pathway. (**O**) Relative expression levels of genes related to the abscisic acid synthesis pathway. *P < 0.05, **P < 0.01, ***P < 0.001

To further understand the molecular mechanisms of allelopathic effects of AAWE, genome-wide gene expression profiles were compared between water- and 0.03 g/mL AAWE- treated rice roots and leaves. After further screening, 689 genes with |Log2FC|≥ 1 and *Q*-value < 0.05 were selected for further investigation (Supplemental Fig. 3A). The clustering heat map is shown in Fig. 6A. There were 311 DEGs in AAWE-treated leaves, of which 245 DEGs were up-regulated, and 66 DEGs were down-regulated. In AAWE-treated root samples, there were 418 DEGs, among which 254 were up-regulated, and 164 were down-regulated. KEGG classification of DEGs showed that most genes were involved in metabolism (Supplemental Fig. 3B). To elucidate DEG functions in response to AAWE stress, the Kyoto Encyclopedia of Genes and Genomes (KEGG) and Gene Ontology (GO) databases were used to enrich DEGs into the corresponding pathways and categories. In leaves, the DEGs were enriched in some important KEGG pathways, such as nitrogen metabolism, photosynthesis, porphyrin, and chlorophyll metabolism (Fig. 6B). Meanwhile, the results of the GO enrichment demonstrated that DEGs were involved in oxidation–reduction processes, transmembrane transport, photosynthesis, and iron ion transport (Fig. 6C). We also analyzed the DEGs between water- and AAWE-treated roots. KEGG pathways related to the biosynthesis of secondary metabolites and starch and sucrose metabolism were identified (Fig. 6D). The GO categories of hydrogen peroxide catabolic process, reactive oxygen species metabolic process, gibberellic acid homeostasis, and iron ion transmembrane transport were also identified (Fig. 6E).

Key driver gene analysis (KDA) was performed to systematically investigate the influence of AAWE on rice plants (Fig. 6F), and ten key driver genes related to the primary metabolism of plants were significantly affected (Supplemental Table 1). Interestingly, all of these genes were involved in starch and sucrose metabolism or galactose metabolism pathways, and some of these genes were linked to chloroplasts. Therefore, we speculated that *A. argyi* allelopathy influenced the primary metabolic processes of rice, such as photosynthesis, and subsequently drove the abnormal expression of other pathways in rice. Furthermore, we performed functional annotation analysis on the related genes linked to the key driver genes (Fig. 6G). Our results demonstrated that genes involved in the starch and sucrose metabolism pathway accounted for 39% of the total, followed by the biosynthesis of secondary metabolites pathway, which accounted for 16%. These results indicated that these related genes were not only related to primary metabolism but were also closely related to secondary metabolism, thus affecting the growth and development of the whole plant.

To validate the transcriptome reliability, RT–qPCR was used to confirm the expression levels of 15 genes involved in the rice response to *A.* argyi allelochemicals. First, the key genes involved in photosynthesis and chlorophyll synthesis pathways in leaves were verified. In our previous study [19], the expression levels of related genes in rice leaves were significantly inhibited after a long treatment time (21 days) with AAWE. In this study, we analyzed the key driver genes of all DEGs between the control and AAWE-treated rice and found that the primary metabolic pathway was the key driver pathway. Therefore, we measured the expression levels of genes related to photosynthesis and chlorophyll synthesis in leaves at an earlier period (13 days) after *A. argyi* stress to detect whether the plant's primary metabolism process was affected during early stages. In the chlorophyll synthesis pathway, *HEML* gene expression was not significantly changed (Supplemental Fig. 4A), and CHLD expression was significantly inhibited at low AAWE concentrations but slightly promoted at 0.05 g/mL (Supplemental Fig. 4B). In addition, the expression of other chlorophyll biosynthesis-related genes gradually decreased as AAWE treatment concentrations increased, including *HEMA*, *CHLH*, *CRD*, *CHLG*, and *CAO,* which indicated that chlorophyll biosynthesis was significantly blocked (Fig. 6H, I, J, K, L). The *PsbY* gene is a member of photosystem II, and *Os04g38410* encodes the subunit of the LHCII complex, which plays a crucial role in photosynthesis. The expression levels of these two genes were significantly down-regulated under AAWE treatment (Fig. 6M, N).

Moreover, the contents of various hormones changed after *A. argyi* treatment (Fig. 3). As such, we also analyzed the expression levels of hormone-related genes in roots. *YUCCA* and *IAA1* were the key genes in the auxin synthesis and signalling pathways, respectively, and their changing trends were consistent. The expression levels of *YUCCA* and *IAA1* in rice treated with low and medium AAWE concentrations gradually decreased, while those in rice treated with high concentrations increased (Supplemental Fig. 4C, 4D). Compared with the blank treatment, the expression level of the auxin signalling pathway-related gene IAA1 significantly increased under 0.05 g/mL treatment (Supplemental Fig. 4C, 4D). Moreover, GA20, a crucial gene in gibberellin synthesis, first decreased and then increased (Supplemental Fig. 4E), while the expression of DELLA, a signalling pathway gene decreased with increasing AAWE concentrations, indicating that the signalling function of gibberellin might be continuously inhibited (Supplemental Fig. 4F). In addition, the expression levels of two genes related to abscisic acid signalling pathway *NCED4* and *PYR* were similar to genes participating in the auxin signalling pathway (Fig. 6O, Supplemental Fig. 4G). Among them, the expression of *NCED4* in rice treated with high AAWE concentrations was significantly higher than that in the blank group (Fig. 6O). These results demonstrate that the expression levels of crucial genes participating in hormone signalling pathways dramatically changed under AAWE treatment.

## Discussion

Carbon nutrition is the basis of plant life. Green plants absorb light energy from the environment through photosynthesis, assimilate carbon dioxide and water, and then produce organic matter to fuel their own growth and development. Therefore, photosynthesis is called "the most important chemical reaction on earth" [34]. Our results showed that "starving" weeds may be one of the key mechanisms of *A. argyi* allelopathy by interfering with photosynthesis. Chloroplasts are easily affected under various abiotic stress [35]. In our study, the chloroplast membrane system was seriously damaged, and the granum thylakoid disappeared in AAWE-treated rice plants, which seriously hindered chloroplast function. When the chloroplast structure is destroyed, chlorophyll synthase activity will be inhibited, and chlorophyll-degrading enzyme activity is enhanced, which will decrease chlorophyll content [36] and result in a series of chain effects. These severe defects further decrease the chlorophyll and soluble sugar contents in AAWE-treated rice plants. In addition, the results of transcriptome sequencing and qPCR showed that the signalling pathways related to photosynthesis, nitrogen metabolism, porphyrin, and chlorophyll metabolism in AAWE-treated leaves were significantly inhibited, further indicating that *A. argyi* allelopathy seriously hinders biomass accumulation and the growth and development of receptor plants.

ROS balance is crucial for plant growth and development. In tissues under oxidative stress, effective ROS control depends on balanced production and elimination rates; the synthesis of soluble antioxidant compounds such as ascorbic acid, vitamin E, and glutathione; and a series of enzymes that neutralize superoxide anions (O^2−^) and hydrogen peroxide (H_2_O_2_), including SOD, CAT, and POD [37]. Nevertheless, AAWE allelopathy leads to ROS burst and membrane system breakdown. Increases in SOD activity can make up for the decline of CAT and POD in rice plants under 0.01 ~ 0.04 g/mL AAWE treatment, which ensures that the ROS content maintains a relatively stable level. However, POD and CAT activities continuously decrease as the AAWE treatment concentration increases, and rice exceeds its tolerance threshold under 0.05 g/mL AAWE treatment, leading to ROS burst and cell damage. Peroxidation can also be reflected by the remarkable increase in MDA content. MDA also reacts with intracellular proteins and nucleic acids, resulting in the loss of function of proteins and nucleic acids, destroying the bridge bond between cellulose molecules, and inhibiting protein synthesis [38]. These results demonstrate that *A. argyi* allelopathy significantly inhibits the function of the antioxidant system in receptor plants. In addition, *A. argyi* allelopathy also breaks hormone balance, affects the process of normal cell division, elongation, and differentiation, and inhibits the absorption of nutrients in the roots of receptor plants. Plant root systems have a specific morphological structure and physiological function, and all changes in cells and organelles in different parts of the root system are responses to external stressors. AAWE has a significantly destructive effect on the cell and organelle structure of the rice root system, including a decrease in root number, hindrance of cell division, exfoliation of the epidermis, disorder of the meristem, degradation of the nucleus, and many black deposits in root tip cells. These abnormal changes hinder the efficiency of root physiological functions, such as absorbing and transferring nutrients. The results based on the detection of mineral elements in rice plants show that the absorption of iron and manganese and the efficiency of calcium and magnesium transfer from roots to leaves significantly decrease after AAWE treatment, which likely further blocks the synthesis of chlorophyll and photosynthesis in plant leaves.

Plant roots are also an important tissue for hormone synthesis. Balanced regulation of endogenous hormones is key for plant growth and development, metabolism, and environmental response. After AAWE treatment, this balance in the rice root system was significantly disturbed. Our results demonstrate that the auxin, gibberellin, and abscisic acid content in rice roots increased as the AAWE concentration increased, including cytokinins as a whole. An excess or lack of hormones will have significant adverse effects on plants. In this study, an overdose of hormone seriously inhibited rice growth, which is consistent with the results of Liu et al. [39]. After allelochemical ferulic acid treatment, the contents of IAA, GA, ZR, and ABA in wheat seedlings accumulated, which inhibited the growth of wheat seedlings and induced the continued increase of ABA content. In summary, *A. argyi* allelopathy induces a rapid increase in endogenous hormone content, which subsequently leads to metabolic disorders. Furthermore, the abnormal physiological structure of rice roots impedes important nutrient absorption process and accelerates plant death.

**Fig. 7:**
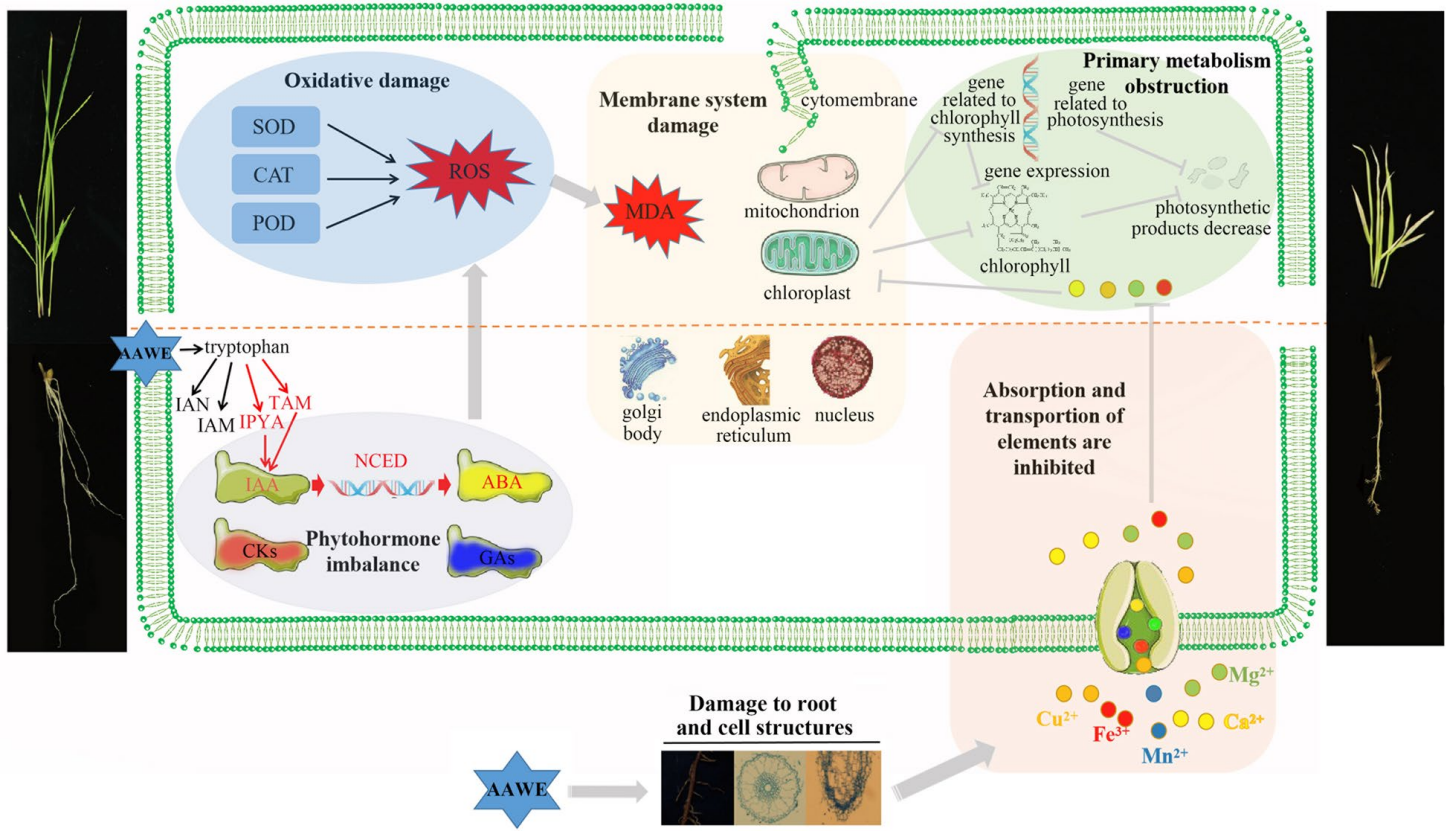
A proposed model of *A. argyi* allelopathy. *A. argyi* realizes its strongly allelopathic effects mainly through the large-scale IAA synthesis and accumulation. The high-intensity stimulation of auxin leads to ROS explosion, damage to membrane system and organelles, and obstruction of ion absorption and transport, all of which ultimately severely inhibit photosynthesis and the primary metabolic processes of receptor plants

Our study on the allelopathic inhibition intensity and related mechanism of *A. argyi* demonstrates that *A. argyi* can be developed as a botanical herbicide. At present, herbicides can be classified according to their mechanism of action, including PSII herbicides, auxin herbicides, HPPD inhibitors, ALS inhibitors, and PPO inhibitors [40]. Among them, many herbicides are widely used in agricultural production. For example, auxin herbicides, mainly represented by phenoxy carboxylic acids, benzoic acids, pyridinecarboxylic acids, aromatic carboxymethyl derivatives, and quinoline carboxylic acids, all have strong herbicidal activity [41]. Low concentrations of auxin in plant tissues can stimulate growth and development processes. However, as auxin concentration and auxin activity increase, plants can be fatally damaged. Since *A. argyi* allelopathy significantly increased the auxin content in tested plants, it might have a similar herbicidal mechanism to auxin herbicides. Regarding the IAA synthesis pathway, plants mainly complete the synthesis of tryptophan to IAA in four ways: the indole pyruvate pathway, tryptamine pathway, indole acetaldehyde oxime pathway, and indole acetamide pathway [42]. In these different synthesis pathways, tryptophan will produce different intermediates under the catalysis of different enzymes to finally form IAA. The indole pyruvate pathway is the basic and primary pathway of auxin synthesis in plants, and the intermediate is indole pyruvate (IPYA). Based on our results, IPYA could not be detected in rice roots under normal growth conditions, showing that this synthetic pathway was hardly active. However, IPYA content increased under AAWE treatment. Similarly, AAWE can strongly activate the tryptamine pathway with tryptamine (TAM) as the intermediate, although it is reported to exist only in a small number of plants. However, the indole acetaldehyde oxime pathway and indole acetamide pathway were inhibited by AAWE. These two pathways coexist in normal-growing rice plants, and the intermediate of the indole acetaldehyde oxime pathway is indole acetonitrile (IAN), which is almost completely inhibited by AAWE. The intermediate of the indole acetamide pathway is indole acetamide (IAM), and AAWE also significantly inhibited this synthesis pathway. The results of this study revealed that AAWE could alter the IAA synthesis pathway in rice and thus leads to the accumulation of large amounts of auxin. It is widely accepted that auxin herbicides cause plants to "grow to death" [43]. The core of this view is that the continuous high-intensity stimulation of auxin in tissues inhibits metabolism and growth and leads to phytotoxic auxin effects. Its metabolic and physiological processes primarily include three stages [41]: the stimulation stage (activation of metabolic process, including leaf abduction, tissue swelling, and stem curl); the greater inhibition stage (root and stem growth inhibition, stomatal closure, decreased carbon assimilation, and starch formation, excessive production of ROS); and the senescence and tissue decay stage (accelerated leaf senescence, chloroplast damage, destruction of membrane, and vascular system integrity). This is consistent with the results of our study; therefore, we consider that AAWE has an herbicidal mechanism similar to that of auxin herbicides (Fig. 7).

A new model for the essential role of ABA accumulation in the synthetic auxin herbicide mode of action has recently been proposed. The rapid up-regulation of *9-cis-epoxycarotenoid deoxygenase* (*NCED*) leads to rapid ABA synthesis and prolonged ABA accumulation, followed by a general inhibition of photosynthesis-related transcription, resulting in leaf senescence, which is the main mode of plant death under auxin herbicide treatment [44]. In our study, the expression of *NCED4* was significantly up-regulated under 0.05 g/mL AAWE treatment, and the ABA content was 3.1 times that of the control. The results of the transcriptional analysis demonstrate that the pathways of chlorophyll synthesis and photosynthesis are inhibited. Therefore, the allelopathic weeding mechanism of *A. argyi* indicates that ABA acts as a second messenger of hormones in the action mode of auxin herbicides [45]. However, this mechanism must be further verified in a follow-up study assessing the allelopathic mechanism of *A. argy*i. On the one hand, if the up-regulation of *NCED* leads to ABA accumulation and down-regulates photosynthesis, it is the key target of auxin herbicide action and plant death. Therefore, the knockout of the *NCED* gene in recipient plants could facilitate the cultivation of herbicide-resistant crops. On the other hand, there is an urgent need for more resources to study the herbicidal and resistance mechanism of these herbicides on weeds, not just in model plants such as rice.

## Conclusions

In conclusion, we conducted a comprehensive and systematic study on the physiological mechanism of *A. argyi* allelopathy and found that AAWE inhibited plant germination and growth via multiple targets and paths. We provide evidence that AAWE can induce the synthesis of large amounts of auxin in plants. This model of inducing target plant hormone imbalance and death through allelopathy is safer than chemical herbicides and produces no residue. Therefore, *A. argyi* can be developed as a plant-derived herbicide, and studies of the physiological mechanism related to *A. argyi* allelopathy also provide a certain theoretical basis for cultivating related resistant crops.

## Methods

### *A. argyi* water extract (AAWE) preparation

The rice seed used in this study were *indica* rice strains, which are widely planted in the middle and lower reaches of the Yangtze River and were purchased from Sichuan Tianyuan Seed Industry Co., LTD (Sichuan, China). *A. argyi* was provided by the Hubei Huichun Qichun Health Industry Development Company (Hubei, China). The desiccative *A. argyi* leaves were smashed with a pulverizer and sifted with a griddle. Leaf powder (125 g) was added to 500 mL of ultrapure water and extracted at room temperature for 48 h with 30 min of ultrasonication every 12 h to obtain an AAWE stock solution (0.25 g/mL). The aqueous solution was filtered through a double filter bag and then stored at 4℃.

### Experimental design: Determination of allelopathy activity of AAWE

Rice seeds were germinated in a filter paper roll soaked with Hoagland nutrient solution containing an AAWE gradient (0, 0.01, 0.02, 0.03, 0.04, 0.05, 0.10, and 0.15 g/mL). Each biological replicate had 10 filter paper rolls placed in culture flasks for culture. Three replicates were performed for each treatment. The experiment was performed in a growing room at a constant temperature of 25 ± 2℃ and a photoperiod of 10 h light/14 h dark. The plant height, root length, and root number of 21 days-old rice plants were measured to evaluate the AAWE allelopathy intensity.

### Microexamination of rice root and leaf system

For microexamination, rice plants were treated with different concentrations (0, 0.01, 0.03, and 0.05 g/mL) of AAWE as described above. The branches of the root system were observed under a stereomicroscope. Then, approximately 0.5 cm root tips of each treatment group were collected and fixed with formaldehyde-acetic acid–ethanol fixative, and then embedded with paraffin. After staining with toluidine blue, longitudinal and cross-sectional root tip cell samples were observed under an IX-73P1F inverted fluorescence microscope (OLYMPUS). SEM was performed as described by Li et al. [46]. The root samples were collected and fixed at 4℃ for 24 h with 2.5% glutaraldehyde (v/v). After fixing, the samples were dehydrated with gradients of 30%, 50%, 70%, and 95% ethanol for 15 min. The last step of dehydration is to stand the sample in absolute ethanol for 45 min. Finally, the dehydrated samples were thoroughly dried in a vacuum dryer and sputter-coated with gold. The processed samples were observed and photographed with a JEOL (JSM-6510LV) under an accelerating voltage of 30 kV [47]. In addition, the ultrastructure of root tip cells was further observed by TEM. Approximately 1 mm root tip was cut and fixed in 2.5% (v/v) glutaraldehyde, the samples were prepared by the State Key Laboratory of Microbiology of Huazhong Agricultural University, and the slices were observed using a HITCHI H-7650 TEM. The ultrastructure of leaf cells was also observed by TEM using the above method.

### ESI-HPLC–MS/MS analysis of phytohormone

Three biological repeats, each consisting of 100 mg (fresh weight) of root samples, were harvested 10 days after treatment with different concentrations (0, 0.01, 0.03 and 0.05 g/mL) of AAWE and used for auxin, CK, GA, and ABA analyses. We used acetonitrile solution to extract the endogenous plant hormones from the samples. The QuEChERS method was used to remove impurities, and the nitrogen purge method was used to concentrate the samples. An AGILENT 1290 high-performance liquid chromatography (Agilent, USA) tandem SCIEX-6500Q trap mass spectrometer (AB, USA) was used to detect the hormone contents. For the measurement of hormones, the peak time and response value of the detected fragment ions were corresponded to the standard substance to identify the hormone type. Finally, the standard curve was used to quantify the detected substances [48].

### Soluble sugar measurement

Extraction and quantification of soluble sugar were performed using the sulfuric acid-anthrone colorimetric method. 100 mg fresh leaf fragments obtained from five concentration treatment plants were ground with 1 mL of ultrapure water and incubated in a water bath at 80 ℃ for 30 min. The supernatant was added to anthrone sulfate solution and reacted at 90 ℃ for 5 min, then analyzed by spectrophotometry at 620 nm. The soluble sugar content was indicated as the percentage of fresh weight.

### Measurement of MDA, ROS, and antioxidant enzyme activities

The MDA content was determined using the thiobarbituric acid (TBA) test. The ROS content was analyzed with a plant ROS enzyme-linked immunosorbent assay (ELISA) kit (Meimian Biotechnology Co. Ltd, China). ELISAs were based on a colorimetric reaction spectrophotometrically determined at 450 nm.

Crude enzyme extracts for SOD, POD, and CAT assays were prepared as follows: fresh leaf samples were homogenized with different extraction reagents (0.9 mL phosphate buffer, 0.1 M pH 7 ~ 7.4, for SOD and CAT determination; 1 mL extraction solvent in the kit for POD determination) in a grinding mill. After centrifugation at 12,000 × g at 4 °C for 10 min, the supernatant was collected for further analysis.

Total SOD activity was assayed by a SOD Assay Kit (Nanjing Jiancheng Bioengineering Institute NJBI). One unit of SOD activity (U) was defined as the amount of enzyme required for the reaction system when the SOD inhibition rate reached 50%. SOD activity was measured by the WST-1 method in units of U/g.

POD activity was assayed by a POD Assay Kit (Beijing Solarbio Science & Technology Co., Ltd.). One unit of POD activity (U) was defined as the 0.01 change per minute of A470 in 1 g of tissue in a 1 mL reaction system. POD activity was measured using the guaiacol method in units of U/g.

CAT activity was assayed by the Catalase (CAT) Assay Kit and Total Protein Quantitative Assay Kit (Nanjing Jiancheng Bioengineering Institute NJBI). One unit of CAT activity (U) was defined as 1 mg of tissue protein consuming 1 µmol of H_2_O_2_ at 405 nm for 1 s. CAT activity was measured by the ammonium molybdate method in units of U/mg prot.

### Elemental analysis in rice

Samples treated with 0 g/mL (CK), 0.01 g/mL (L), 0.03 g/mL (M), and 0.05 g/mL (H) AAWE for 21 days were selected to analyze the absorption of mineral elements in rice under allelopathic stress. All of the plant tissue (roots and leaves) was heated at 105 ℃ for 15 min before being dried at 60 ℃. The dried samples were weighed for elemental analysis. Then, dried samples were ground and digested with HNO_3_-HF (10 mL-1 mL) using a microwave digestion apparatus. After removing nitric acid from the acidometer (135 ℃ 2.5 h), ultrapure water was added to reach the final volume (50 mL) [49]. The elements, including K, Ca, Mg, Fe, Mn, and Cu, were measured by atomic absorption spectrophotometry (Persee A3, China), and P was measured by molybdenum antimony colorimetry. 0.10 g dried samples were accurately weighted. After adding 8 mL H_2_SO_4_, 3 g K_2_SO_4_, and 0.2 g CuSO_4_, the samples were placed on a graphite digestion apparatus for digestion (180 ℃ for 30 min; 280 ℃ for 30 min; 420 ℃ for 60 min). And the N content was then determined using the Kjeldahl method [49]. Three biological replicates were performed for each treatment group.

### Transcriptome analysis

For RNA-seq, three independent biological replicates were performed. Rice plants were treated with blank solution (CK-L, CK-R) and 0.03 g/mL AAWE (T-L, T-R) for 7 days, and then the leaf and root samples were collected. CTAB-PBIOZOL reagent was used to purify total RNA [50]. The sequencing was performed on the BGISEQ500 platform at the Beijing Genomics Institute (BGI) in Shenzhen. Clean RNA-seq reads were mapped to the rice reference genome. The expression level of the gene was calculated by RSEM (v1.2.12) [51]. And a threshold analyzed by DESeq2 (v1.4.5) [52] of Q value ≤ 0.05 was used to determine the differential expression. The DEGs were used for GO and KEGG (www.kegg.jp/kegg/kegg1.html) [53] enrichment analyses according to the hypergeometric test by Phyper.

### Real-time quantitative PCR (RT–qPCR)

After 13 days of AAEW treatment, the expression patterns of nine genes associated with photosynthesis and chlorophyll synthesis pathways related to leaves and nine genes associated with hormone synthesis and signalling pathways related to roots were analyzed using RT-qPCR (gene-specific primers are listed in Supplemental Table 2). Total RNA was isolated from the leaves and roots using TRIzol (Tiangen Bio Co., Ltd., Beijing, China), and the cDNA was synthesized using MLV reverse transcriptase (Promega Corporation, USA). RT–qPCR was conducted using RealUniversal Colour PreMix (SYBR Green) according to the manufacturer’s instructions (Tiangen, Beijing, China). The *ACTIN* gene was used as an internal standard. The relative gene expression level of each target gene was determined using the 2^−ΔΔt^ method [19].

## Authors’contributions

DL and JL designed the research project. JL, TZ, LC, HC, DL and CC performed most of the experiments. LD and YM supervised the experiments. JL wrote the article. LD and YM supervised and complemented the writing of the article.

## Supplementary Information

**Additional file 1: Figure S1. **Roots were destroyed in AAWE-treated rice. **Figure S2. **The AAWE-treated rice leaves were abnormal. **Figure S3. **Preliminary analysis of DEGs between control and AAWE-treated rice. **Figure S4.** Relative expression levels of genes in rice leaves and roots under AAWE treatment. **Table S1. **Details of key driver genes. **Table S2. **The sequence of gene-specific primers for RT-qPCR analysis. 

## Data Availability

Data supporting the finding of this study are available within the article and its supplementary files. The raw transcriptome data have been submitted to NCBI SRA under the project number: PRJNA850081 (https://www.ncbi.nlm.nih.gov/bioproject/ PRJNA850081).
