# Supplementary material for: Artemisia argyi allelopathy: a generalist compromises hormone balance, element absorption, and photosynthesis of receptor plants
Source: BMC Plant Biol. 2022 Jul 26;22:368. doi: 10.1186/s12870-022-03757-9 (PMC9317128; doi:10.1186/s12870-022-03757-9)
Supplement: Supplementary file 1 — Additional file 1: Figure S1. Roots were destroyed in AAWE-treated rice. Figure S2. The AAWE-treated rice leaves were abnormal. Figure S3. Preliminary analysis of DEGs between control and AAWE-treated rice. Figure S4. Relative expression levels of genes in rice leaves and roots under AAWE treatment. Table S1. Details of key driver genes. Table S2. The sequence of gene-specific primers for RT-qPCR analysis. [file 12870_2022_3757_MOESM1_ESM.pdf]

SUPPORTING INFORMATION

Artemisia argyi allelopathy: a generalist compromises hormone balance, element absorption and photosynthesis of receptor plants

Jinxin Li[†], Tingting Zhao[†], Le Chen, Hong Chen, Dandan Luo, Changjie Chen, Yuhuan Miao* and Dahui Liu*

Key Laboratory of Traditional Chinese Medicine Resources and Chemistry of Hubei Province, Hubei University of Chinese Medicine, Wuhan 430065, China

* For correspondence (e-mails liudahui@hbtcm.edu.cn; miaoyh@hbtcm.edu.cn).

† These authors contributed equally to this work.

Figure S1

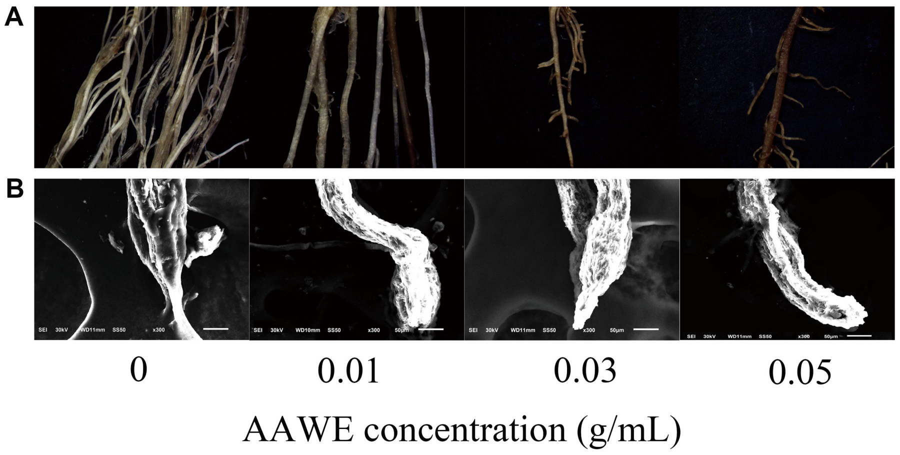

Figure S1. Roots were destructed in AAWE-treated rice.

(A) Root development and branching under different treatment conditions.

(B) Scanning electron microscopy of wild-type and AAWE-treated root tips. Scale bars, 50 μm.

Figure S2

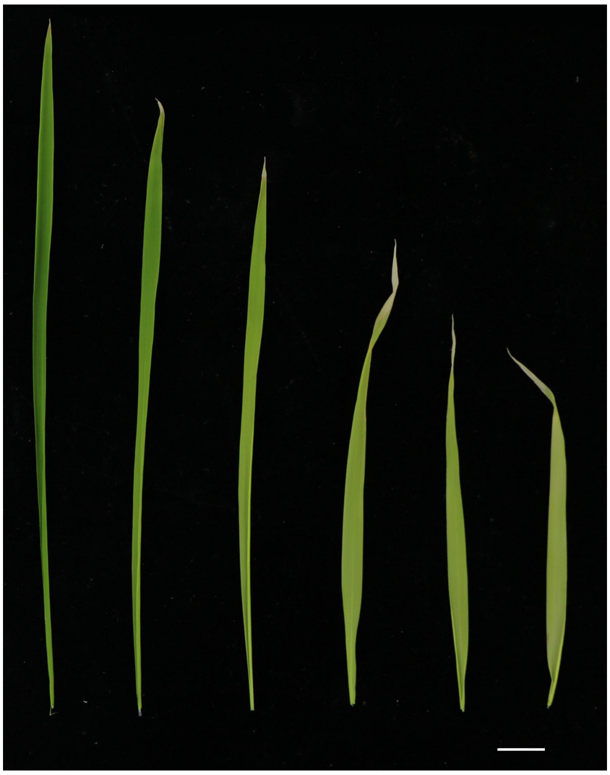

Figure S2. The AAWE-treated rice leaves were abnormal.

Leaf rolling, yellowing and withering occurred in AAWE-treated rice leaves. The leaves from left to right showed control, 0.01 g/mL, 0.02 g/mL, 0.03 g/mL, 0.04 g/mL, 0.05 g/mL AAWE treatment. Scale bar, 1 cm.

Figure S3

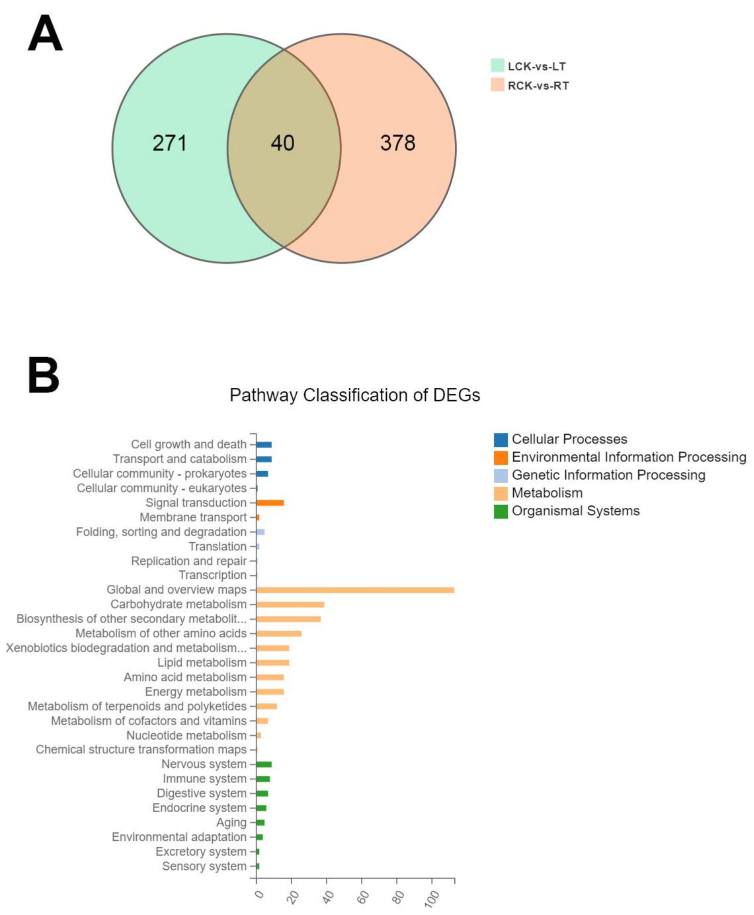

Figure S3. Preliminary analysis of DEGs between control and AAWE-treated rice.

(A) The number of DEGs distributed in leaves and roots after screening (|Log2FC|≥1).

(B) KEGG classification results of DEGs.

Figure S4

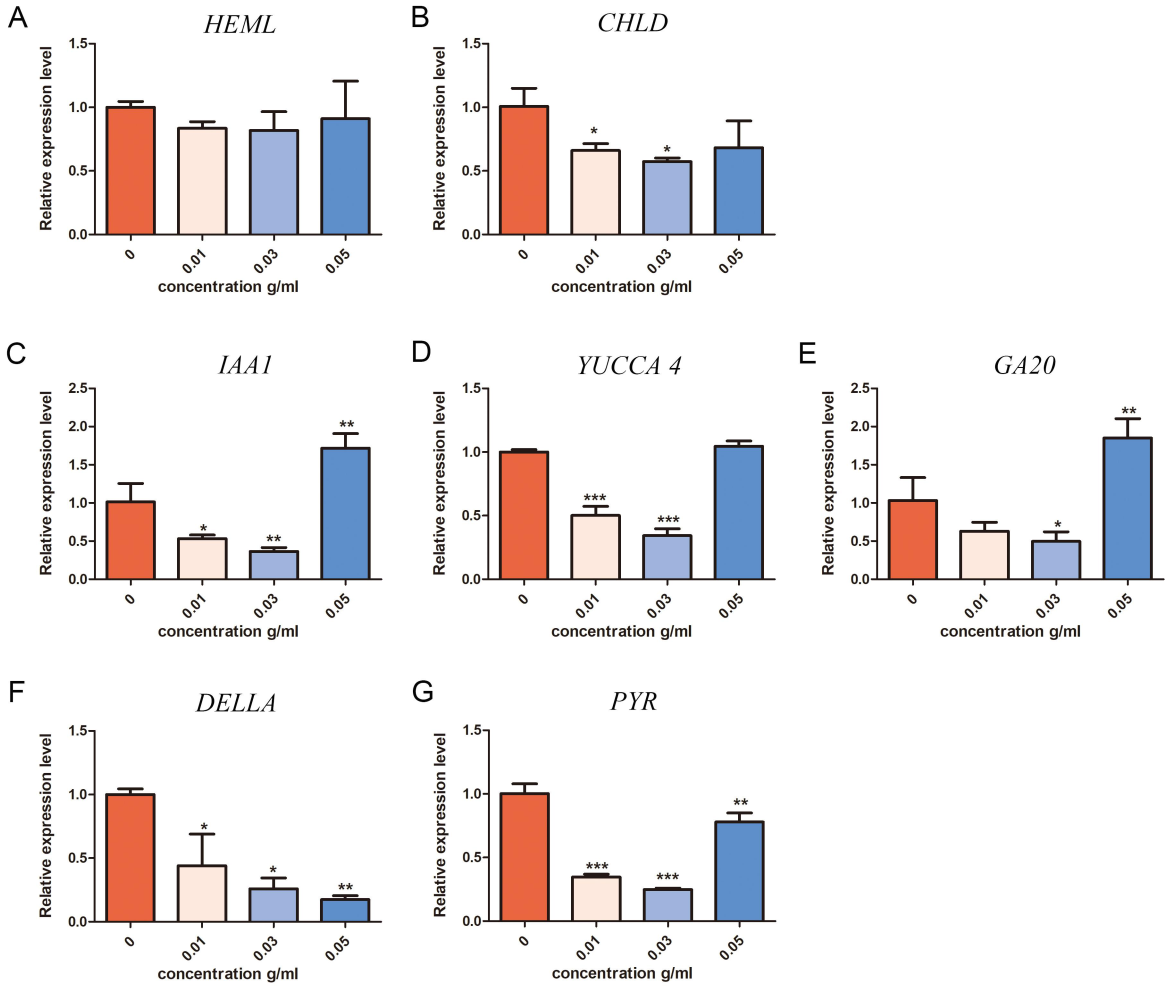

Figure S4. Relative expression levels of genes in rice leaves and roots under AAWE treatment.

(A-B) Genes that are not differentially expressed related to the chlorophyll synthesis pathway in control and AAWE-treated leaves.

(C-G) Expression levels of hormone-related genes in roots. C, *IAA1*, related to auxin synthesis; D, *YUCCA4*, related to auxin signalling pathways; E, *GA20*, related to gibberellin synthesis; F, *DELLA*, related to gibberellin signalling pathways; G, *PYR*, related to abscisic acid signalling pathways.

Table S1. Details of key driver genes.

Gene ID	Gene Symbol	Describe	Function comment information	
			KEGG Pathway	GO Biological Process
LC4342117	LOC_Os06g51084	1,4-alpha-glucan-branching enzyme, chloroplastic/amyloplastic-like	Starch and sucrose metabolism	carbohydrate metabolic process<br>glycogen biosynthetic process
LC4333062	LOC_Os03g28330	sucrose synthase 1-like	Starch and sucrose metabolism	sucrose metabolic process<br>response to carbon dioxide
LC4343045	LOC_Os07g23944	alpha-glucosidase 2	Galactose metabolism<br>Starch and sucrose metabolism	carbohydrate metabolic process
LC4331252	LOC_Os02g58480	sucrose synthase 6-like	Starch and sucrose metabolism	sucrose metabolic process
LC4329561	LOC_Os02g33110	beta-fructofuranosidase, insoluble isoenzyme 1-like	Galactose metabolism<br>Starch and sucrose metabolism	carbohydrate metabolic process
LC4335447	LOC_Os04g24430	sucrose synthase 5-like	Starch and sucrose metabolism	sucrose metabolic process
LC4332788	LOC_Os03g22120	sucrose synthase 4-like	Starch and sucrose metabolism	sucrose metabolic process
LC4348840	LOC_Os10g32810	beta-amylase 1, chloroplastic-like	Starch and sucrose metabolism	polysaccharide catabolic process
LC4331577	LOC_Os03g04770	beta-amylase 2, chloroplastic-like	Starch and sucrose metabolism	polysaccharide catabolic process
LC4335790	LOC_Os04g33740	beta-fructofuranosidase, insoluble isoenzyme 2-like	Galactose metabolism<br>Starch and sucrose metabolism	carbohydrate metabolic process<br>cell proliferation<br>defense response to bacterium<br>leaf development<br>cotyledon development<br>defense response to fungus<br>carbohydrate storage

Table S2. The sequence of gene-specific primers for RT-qPCR analysis.

Primer name	Primer sequence	Description
Actin-F	TGTAAGCAACTGGGATGA	RT-qPCR
Actin-R	CCTTCGTAGATTGGGACT	
HEMA-F	GATGCAATCACTGCTGGAAAGCGT	RT-qPCR
HEMA-R	CCATCTTGCCAGCACCAATCAACA	
HEML-F	AGAACAAAGGGCAGATTGCTGCTG	RT-qPCR
HEML-R	TGTTTCGTCAAGTCACGGAGAGCA	
CHLD-F	TAGCACAGCTGTCAGAGTGGGTTT	RT-qPCR
CHLD-R	TTGCCAGCCACCTCAAGTATCTCA	
CHLH-F	GCACGGGAACTTGGCGTTTCATTA	RT-qPCR
CHLH-R	ACATGTCCTGGAGCTGCTTCTCAT	
CRD-F	TGGATCTAACATGACACGCACCCA	RT-qPCR
CRD-R	ACTGTAACGGCATTCTTCTCCGGT	
CHLG-F	CCAGCCACTGATGAAAGCAGCAAT	RT-qPCR
CHLG-R	AGAGCGCTAATACACTCGCGAACA	
CAO-F	ACACCTTCATCTGGGCTTCAAGGA	RT-qPCR
CAO-R	AGATGCGTCGAACATTGCTTGGTG	
PsbY-F	CGCAGCGACATTGCACTCACC	RT-qPCR
PsbY-R	TGACGCCTTCTTCTTCCTCCTCTT	
Os04g38410-F	TGACGCCTTCTTCTTCCTCCTCTT	RT-qPCR
Os04g38410-R	GCTACTGCTCCTCCGACCTCCAAT	
YUCCA4-F	GGCCGGCCTAATTTTAACTGG	RT-qPCR
YUCCA4-R	TTCTCAAAAAAGACGCGCGAC	
IAA1-F	GCCGCTCAATGAGGCATT	RT-qPCR
IAA1-R	GCTTCCACTTTCTTTCAATCCAA	
GA20-F	GCCACTACAGGGCCGACAT	RT-qPCR
GA20-R	TGGTTGCAGGTGACGATGAT	
DELLA-F	CTACTACTCCACCATGTTCGAT	RT-qPCR
DELLA-R	CTGTTTGTAGGCATTGGAGC	
NCDE4-F	GATTGCACGGCACCTTCATT	RT-qPCR
NCDE4-R	CTCTGTAATTTGATTTTTCACTGGCTAAT	
PYR-F	GTAGTTCCTCCATTGTCTTC	RT-qPCR
PYR-R	GACCTAAGCCCATCACTA	
